# Osmotic Stress Confers Enhanced Cell Integrity to Hydrostatic Pressure but Impairs Growth in *Alcanivorax borkumensis* SK2

**DOI:** 10.3389/fmicb.2016.00729

**Published:** 2016-05-18

**Authors:** Alberto Scoma, Nico Boon

**Affiliations:** Center for Microbial Ecology and Technology, Department of Biochemical and Microbial Technology, University of GhentGhent, Belgium

**Keywords:** osmolyte, piezolyte, hydrocarbons, oil, deep-sea, ectoine, *Halomonas*, petroleum

## Abstract

*Alcanivorax* is a hydrocarbonoclastic genus dominating oil spills worldwide. While its presence has been detected in oil-polluted seawaters, marine sediment and salt marshes under ambient pressure, its presence in deep-sea oil-contaminated environments is negligible. Recent laboratory studies highlighted the piezosensitive nature of some *Alcanivorax* species, whose growth yields are highly impacted by mild hydrostatic pressures (HPs). In the present study, osmotic stress was used as a tool to increase HP resistance in the type strain *Alcanivorax borkumensis* SK2. Control cultures grown under standard conditions of salinity and osmotic pressure with respect to seawater (35.6 ppt or 1136 mOsm kg^-1^, respectively) were compared with cultures subjected to hypo- and hyperosmosis (330 and 1720 mOsm kg^-1^, or 18 and 62 ppt in salinity, equivalent to brackish and brine waters, respectively), under atmospheric or increased HP (0.1 and 10 MPa). Osmotic stress had a remarkably positive impact on cell metabolic activity in terms of CO_2_ production (thus, oil bioremediation) and O_2_ respiration under hyperosmosis, as acclimation to high salinity enhanced cell activity under 10 MPa by a factor of 10. Both osmotic shocks significantly enhanced cell protection by reducing membrane damage under HP, with cell integrities close to 100% under hyposmosis. The latter was likely due to intracellular water-reclamation as no trace of the piezolyte ectoine was found, contrary to hyperosmosis. Notably, ectoine production was equivalent at 0.1 MPa in hyperosmosis-acclimated cells and at 10 MPa under isosmotic conditions. While stimulating cell metabolism and enhancing cell integrity, osmotic stress had always a negative impact on culture growth and performance. No net growth was observed during 4-days incubation tests, and CO_2_:O_2_ ratios and pH values indicated that culture performance in terms of hydrocarbon degradation was lowered by the effects of osmotic stress alone or combined with increased HP. These findings confirm the piezosensitive nature of *A. borkumensis*, which lacks proper resistance mechanisms to improve its metabolic efficiency under increased HP, thus explaining its limited role in oil-polluted deep-sea environments.

## Introduction

*Alcanivorax* is a marine hydrocarbonoclastic genus, which dominates oil-polluted surface waters worldwide (Syutsubo et al., 2001; Hara et al., 2003; Yakimov et al., 2005). Its negligible presence in deep-sea areas contaminated with hydrocarbons (e.g., the Gulf of Mexico, following the deepwater Horizon (DWH) oil spill accident; Hazen et al., 2010; Valentine et al., 2010; Baelum et al., 2012; Mason et al., 2012; Gutierrez et al., 2013; Yergeau et al., 2015) supported the hypothesis that this genus might lack proper adaptation mechanisms to hydrostatic pressure (HP). Recent laboratory experiments on *A. jadensis* and *A. dieselolei* strains subjected to HP up to 10 MPa (equivalent to 1 km depth, approximately the depth of the oil plume formed after the DWH spill, Camilli et al., 2010) supported this hypothesis (Scoma et al., 2016). Similar tests on the type strain *Alcanivorax borkumensis* SK2 further refined these results, and indicated that the expression of all the genes related with the biosynthetic pathway of ectoine and its actual production per cell are enhanced under 10 MPa (Scoma et al., unpublished results). The mechanisms by which ectoine might offset HP remain unclear, as well as its possible role in supporting fitness, cell replication, or functionality.

Ectoine was first isolated from the halophilic phototrophic bacterium *Ectothiorhodospira halochloris* (Galinski et al., 1985) to which it owes its trivial name, and early after detected in several other microbes such as some *Halomonadaceae* (Wohlfarth et al., 1990), Bacterium Ba_1_, *Vibrio costicola* (Regev et al., 1990) and *Brevibacterium linens* (Bernard et al., 1993). Intracellular accumulation was noted to be proportional to an increase in osmolarity (Galinski et al., 1985) and could result from *de novo* synthesis using glutamate as precursor (Inbar and Lapidot, 1988) or uptake from the outer environment (Jebbar et al., 1992). Nonetheless, its intracellular concentration could be negatively regulated by supplying cells with other osmolytes or their precursors (e.g., glycine betaine, choline, proline, trehalose, and taurine) likely owing to ectoine energy-intensive *de novo* biosynthesis (Bernard et al., 1993). First evidence of its transcriptional regulation was found in *Halomonas elongata*, where a 3-gene cluster was identified (*ectABC*, Cánovas et al., 1997, 1998) and soon discovered to be evolutionary highly conserved and widespread (Louis and Galinski, 1997; Göller et al., 1998; Pflughoeft et al., 2003; Reshetnikov et al., 2006; Bursy et al., 2007; Saum and Müller, 2008). In recent years, other mechanisms have been found to account for the enhanced transcription of *ectABC*, such as high (Calderon et al., 2004) and low temperature (in *Virgibacillus pantothenticus*, Kuhlmann et al., 2008) and, as previously mentioned, HP (in *A. borkumensis* SK2, Scoma et al., unpublished results).

Intracellular accumulation of inorganic and organic compounds to counteract environmental osmotic stress is a widespread response in the microbial world (Sleator and Hill, 2002). Microorganisms can synthesize or take up such compounds from the environment to prevent cell disruption and/or other water-related stresses, which may co-occur with a change in osmolarity (e.g., oxidation, protein perturbation; Yancey, 2005). Although, much remains to be understood about the relationship among solute-accumulation, water retention and protein functionality, it appears that several osmolytes accumulated within the cell exert a protective function also with respect to other extreme conditions [e.g., high or low temperature and pH, toxic compounds (Wood, 1999; Welsh, 2000)]. This observation has been also reported for HP. Increased concentration of sodium chloride or sugars in several different microbes reduced the inactivation rate to high HP (>200 MPa; Oxen and Knorr, 1993; Palou et al., 1997; Molina-Höppner et al., 2004). The protective effect provided by osmotic stress response to enhanced HP may differently impact cell physiology and integrity. In *Lactococcus lactis*, supplying 0.5 M sucrose preserved metabolic activity and membrane integrity of cells exposed up to 600 MPa HP, whereas inorganic salts (4 M NaCl) preserved cell membrane integrity but failed to have a positive impact on cell metabolism (Molina-Höppner et al., 2004). In *A. borkumensis* SK2, application of 5 MPa essentially inactivated cultures supplied with *n*-dodecane, showing a net decrease in cell number and only 10% membrane integrity in the surviving cells (Scoma et al., unpublished results). However, incubation at 10 MPa re-established cell growth and nutrient uptake capacity, significantly enhanced cell integrity (∼25%) and resulted in a remarkable intracellular ectoine accumulation (Scoma et al., unpublished results). Another *Alcanivorax* species subjected to the same conditions (i.e., *A. dieselolei*, 10 MPa, *n*-dodecane as sole carbon source) showed much higher cell integrity levels at 10 MPa (∼70%) and no ectoine accumulation or gene expression upregulation (Scoma et al., 2016) suggesting a possible relationship between cell membrane integrity and intracellular ectoine levels at increased HP. In order to understand the physiological conditions triggering ectoine accumulation, in the present investigation different ectoine intracellular levels were stimulated by altering the physiological parameters known to affect its synthesis, that is, osmolarity and HP. *A. borkumensis* SK2 control cultures grown under standard isosmotic conditions similar to seawater were compared with cultures acclimated to altered osmotic conditions, the salinity of which resembled brackish or brine waters. All cultures were subjected to either 0.1 or 10 MPa HP. Results aimed primarily at testing whether shifts in extracellular osmolarity might protect *Alcanivorax* cells from the damaging effects exerted by enhanced HP and were discussed with respect to the response in ectoine accumulation.

## Materials and Methods

### Strain, Standard Medium, and Growth Conditions

*Alcanivorax borkumensis* SK2 was cultivated axenically in Schott glass bottles of 250 mL (operating volume 100 mL), without providing mixing, under ambient pressure (i.e., 0.1 MPa), using ONR7a medium (Dyksterhouse et al., 1995), initial pH 7.6, for 4–7 days at 20°C. Cultures were provided with 1% (v:v) *n*-dodecane (Sigma-Aldrich, Belgium) as sole carbon source (equivalent to about 7.5 g L^-1^) to imitate the conditions of an oil spill (high C/N ratio) as previously suggested for this strain (Sabirova et al., 2006).

### Modified Media and Growth Conditions

Sea and ocean water are typically defined as saline waters, and have an average osmolarity of about 1000 mOsm kg^-1^ (Yancey, 2005) equivalent to a salinity of 35 ppt (or g kg^-1^) or, alternatively, 3.5% (**Table 1**). The large majority of the salinity in seawater is determined by NaCl (Yancey, 2005). The ONR7a medium is a standard medium to cultivate marine bacteria, has a salinity of 35.6 ppt, equivalent to 1136 mOsm kg^-1^ (**Table 1**) and is therefore isosmotic with respect to seawater (osmolarity was calculated as the sum of the osmotic pressure of all the single salts used to prepare the medium). In the present study, modified osmolarity was achieved by targeting the two most concentrated salts exerting the highest osmotic pressure in the ONR7a medium. The possibility of using proportional concentrations of the entire ONR7a medium was discarded, as altering the concentration of all the elements in seawater would have little effect on the osmolarity, while potentially impacting the whole microbial metabolism. The two most concentrated salts in ONR7a, accounting for about 90% of ONR7a osmotic pressure, are NaCl and MgCl_2_ (780 and 165 mOsm kg^-1^, respectively). Selection of another salt in combination with NaCl was necessary to impose a stronger hyposmotic effect to counteract HP by means of water influx into the cell, provided that a reduction of NaCl from 22.8 to 8.2 g L^-1^ would still yield an osmotic pressure of 638 mOsm kg^-1^ (vs. 1136 mOsm kg^-1^ in standard ONR7a). In moderately halophilic bacteria, several ions other than Na^+^ or Cl^-^ are known to play a role in osmotic equilibrium, such as K^+^, Mg^2+^, Ca^2+^, and Mn^2+^ (Ventosa et al., 1998). In the ONR7a medium, MgCl_2_ was preferred over KCl because it brings a higher osmotic pressure in standard formulations (165 vs. 19.3 mOsm kg^-1^, respectively). NaCl and MgCl_2_ are normally provided in ONR7a at concentrations of 22.8 and 11.18 g L^-1^, respectively. Final concentrations under hyposmosis were 8.2 and 4.7 g L^-1^, while under hyperosmosis they were 44 and 21.5 g L^-1^, respectively. Hence, hypo-, iso-, and hyperosmosis exerted a different final osmotic pressure on bacterial cells equivalent to 330, 1136, and 1720 mOsm kg^-1^ (**Table 1**). In other words, the marine bacterium *A. borkumensis* SK2 was tested using very different salinities, equivalent to brackish, saline, and brine waters, respectively.

**Table 1:** Overview of the different media salinities and osmotic pressures with respect to seawater.

Culture conditions	Medium	Definition	Salinity		NaCl	MgCl	Osmotic pressure	Reference
			ppt *or* g kg^-1^	%	*M*	*M*	mOsm kg^-1^	
Isosmosis	Seawater	Saline	35.0	3.5	0.47	0.07	1000	Yancey, 2005
Isosmosis	ONR7a	Saline	35.6	3.6	0.39	0.05	1136	Dyksterhouse et al., 1995
Hyposmosis	Modified ONR7a	Brackish	18.0	1.8	0.03	0.02	330	This work
Hyperosmosis	Modified ONR7a	Brine	61.7	6.2	0.75	0.11	1720	This work

To achieve acclimation to a different salinity/osmolarity, SK2 cells were first incubated for 7–10 days using media at altered osmotic conditions. Then, 10% culture volume was withdrawn and re-incubated using the same modified ONR7a medium (either hypo- or hyperosmotic) for another 7–10 days of incubation. Cultures were considered acclimated to the new osmolarity after completing three full cycles of growth in their respective modified ONR7a. Later, acclimated cells were used in specific tests under ambient and increased HP. All other culture conditions were the same as for isosmosis.

### Ambient and Increased HP Experiments

Cells were collected by centrifugation at 4000 rpm for 10 min at 4°C (Sorval RC5c PLUS, Beckman, Suarlée, Belgium), and resuspended in standard or modified ONR7a medium at an initial optical density (OD_610_) of 0.33 ± 0.02, corresponding to 3.6 ± 0.2 × 10^8^ cells mL^-1^. Cells grown under isosmotic conditions were tested using standard ONR7a (isosmosis) or modified ONR7a exerting a different osmotic pressure (hyposmosis or hyperosmosis). Cells acclimated to a different osmolarities were tested using their respective medium (acclimated hyposmosis or hyperosmosis). None of the cultures was adapted to HP. Culture suspensions (3.5 mL) were transferred into sterile 10 mL syringes, and *n*-dodecane (C12) 1% (v:v) was supplied as the sole carbon source. The gas phase (equal to 6.5 mL) was constituted of air, which provided O_2_ to the cells during the subsequent incubation. Syringes were closed using a sterile three-way valve, and placed in a 1 L T316 stainless steel high-pressure reactor (HPR; Parr, USA). The reactor was filled with deionized water and HP was increased up to 10 MPa (equivalent to 1000 m below surface level) by pumping water with a high-pressure pump (HPLC pump series III, SSI, USA). Pressure was transmitted to the cultures through the piston of the syringe. Experiments at atmospheric pressure were run adjacent to the HP reactor. Control experiments were conducted using sterile syringes supplied only with sterile non-inoculated medium to check on CO_2_ and O_2_ levels in the headspace at both 0.1 and 10 MPa. Incubations were conducted in a temperature-controlled room at 20°C for 4 days. At the end of the experiments, HP was gently released and syringes set aside for 30 min before running biochemical analyses.

To assess the metabolic activity, efficiency, and oil bioremediation potential of *A. borkumensis* cultures, biomass growth, CO_2_ production, CO_2_:O_2_ ratios and pH decrease were measured at the end of the incubations. Cell membrane integrity was considered as a primary target for cell viability under stressing conditions, provided that both hydrocarbons (Heipieper and Martinez, 2010) and HP (Molina-Höppner et al., 2004) are known to impact this parameter, and its potential correlation with ectoine accumulation considered (Scoma et al., unpublished results).

### Cell Counts and Integrity

Pressure-induced cell membrane damage analysis (Benito et al., 1999) and total cell count was done using flow cytometry: SYBR^®^ Green I and Propidium Iodide were used in combination to discriminate cells with intact and damaged cytoplasmic membranes using the protocol described by De Roy et al. (2012). CO_2_ production and O_2_ respiration per cell were measured considering final cell numbers.

### Chemical Analyses

O_2_ respiration and CO_2_ production rates were assessed by analyzing the headspace gas composition of syringes inoculated with strain SK2 cells as compared to sterile controls. The gas-phase was analyzed with a Compact GC-TCD (Global Analyser Solutions, Breda, The Netherlands), equipped with a Molsieve 5A pre-column and two channels (channel 1 for CH_4_, O_2_, H_2_ and N_2_ and channel 2 for CO_2_, N_2_O, and H_2_S). pH was measured using a pH meter (Herisau, Metrohm, Switzerland). Intracellular ectoine concentration was measured by collecting cells by centrifugation at 4°C (10000 *g*, 5 min), discarding the supernatant and storing pellets at -20°C. Later on, ectoine was extracted from pellets with an ethanol:water solution (4:1, v/v) for 30 min. Following centrifugation, the supernatant was evaporated overnight at 40°C and the residue dissolved in distilled water. Re-suspended ectoine in water was analyzed by HPLC using an Aminex HPX-87C column (Bio-Rad Laboratories, Hercules, CA, USA), with CaCl_2_ (5 mM) as eluent and detection at 210 nm (Onraedt et al., 2004).

### Statistical Analysis

Results were expressed as mean values of experiments made in 10 independent replicates. Error bars in the graphs indicate a 95% confidence interval (95% CI) calculated using a Student *t*-test with a two-sided distribution. Statistical significance was assessed using a non-parametric test (Mann–Whitney test) which considered a two-sided distribution with 95% CI.

## Results

### Hyperosmosis Improves Cell Activity at Ambient and Increased HP

*Alcanivorax borkumensis* SK2 cells where incubated at different osmotic (hypo-, iso- or hyperosmosis, equivalent to 330, 1136, and 1720 mOsm kg^-1^ respectively) and HP (0.1 or 10 MPa) conditions to test whether the response to osmotic stress could prevent or mitigate the negative impact of HP on cell metabolism and culture performance.

**FIGURE 1:**
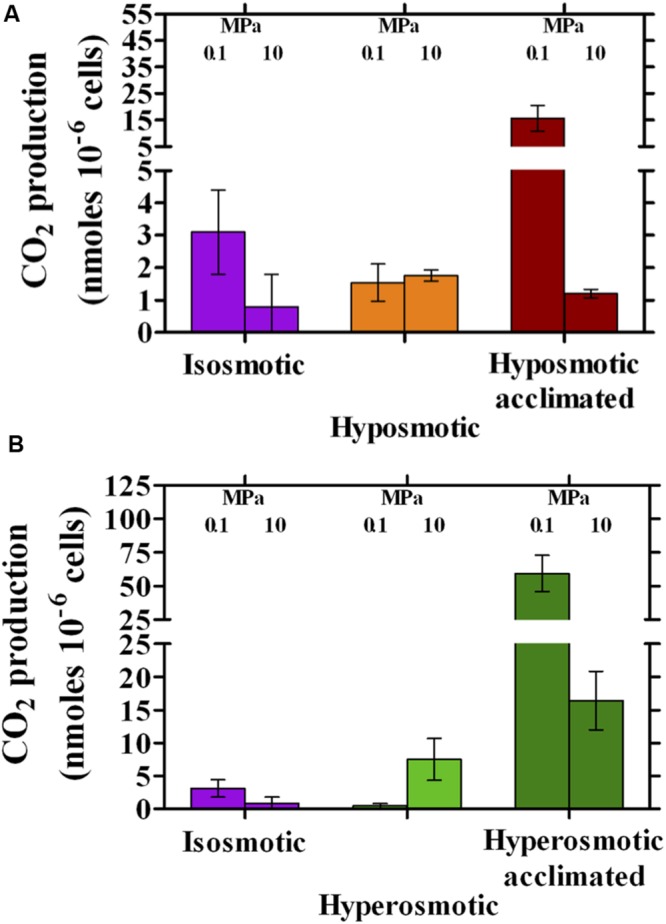
**Production of CO_2_ per cell by *Alcanivorax borkumensis* SK2 cells subjected to hyposmotic **(A)** or hyperosmotic **(B)** conditions under different hydrostatic pressures (0.1 or 10 MPa), as compared to standard isosmotic conditions.** Keys: isosmosis (purple), hyposmosis (orange), hyposmosis acclimated (red), hyperosmosis (light green), hyperosmosis acclimated (dark green). Bars indicate 95% confidence intervals.

Cell metabolism was highly stimulated by a change in osmolarity and/or HP (**Figure 1**). Provided that *n*-dodecane was supplied as sole carbon source, CO_2_ production was considered as an indirect measure of its oxidation by *A. borkumensis*. Under isosmotic conditions at 0.1 MPa, SK2 produced 3.1 ± 1.3 nmoles CO_2_ 10^-6^ cells but faced a threefold reduction in activity when incubated at 10 MPa (**Figure 1A**). Transition to hyposmosis resulted into similar CO_2_ outputs at 0.1 and 10 MPa, which were of the same order as those observed under isosmotic conditions (∼1.5 nmoles CO_2_ 10^-6^ cells, **Figure 1A**). Acclimation to hyposmosis at 0.1 MPa eventually resulted into a 10-fold increase (15.6 ± 4.8 nmoles CO_2_ 10^-6^ cells, **Figure 1A**) while no effect could be observed at 10 MPa after acclimation. Thus, at ambient pressure acclimation to hyposmosis eventually enhanced cell metabolism, while a concomitant reduction in osmolarity and increase in HP prevented this effect (**Figure 1A**).

Conversely, hyperosmosis generally enhanced CO_2_ production. At ambient pressure, transition to a hyperosmotic environment first showed a strong negative impact on CO_2_ production (from 3.1 ± 1.3 to 0.41 ± 0.38 nmoles CO_2_ 10^-6^ cells, **Figure 1B**) while acclimation resulted into a notable increase (59 ± 13 nmoles CO_2_ 10^-6^ cells, **Figure 1B**), which was the highest of all the tested conditions. A positive correlation between increased osmolarity and HP was also observed, as CO_2_ production at 10 MPa increased remarkably from iso- to hyperosmosis (from 0.8 ± 1.0 to 7.5 ± 3.2 nmoles CO_2_ 10^-6^ cells, respectively, **Figure 1B**), with acclimation to hyperosmosis yielding a further improvement to 16.4 ± 4.5 nmoles CO_2_ 10^-6^ cells (**Figure 1B**).

**FIGURE 2:**
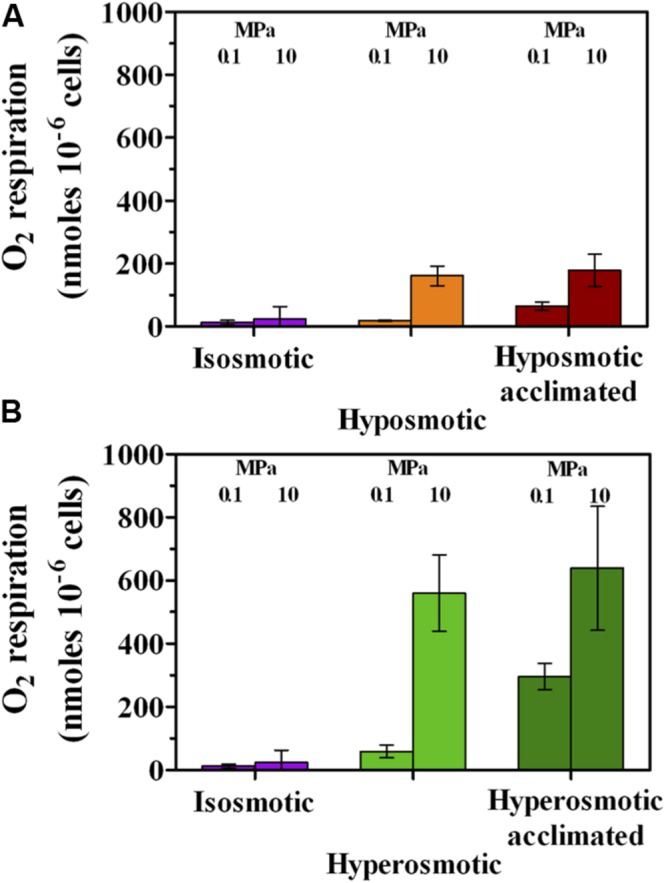
**O_2_ respiration per cell by *A. borkumensis* SK2 cells subjected to hyposmotic **(A)** or hyperosmotic **(B)** conditions under different hydrostatic pressures (0.1 or 10 MPa), as compared to standard isosmotic conditions.** Keys: isosmosis (purple), hyposmosis (orange), hyposmosis acclimated (red), hyperosmosis (light green), hyperosmosis acclimated (dark green). Bars indicate 95% confidence intervals.

In agreement, O_2_ respiration was stimulated more by hyper- rather than hyposmosis (**Figure 2**). Under atmospheric pressure, hyposmosis slightly enhanced O_2_ respiration only after acclimation (**Figure 2A**). On the contrary, the concomitant application of increased HP and hyposmosis resulted into a prompt increase from 24 ± 39 to 161 ± 31 nmoles O_2_ 10^-6^ cells, which remained constant also after acclimation (**Figure 2A**). With regard to hyperosmosis, at 0.1 MPa it increased O_2_ respiration by a factor of 5 (13, 59, and 296 nmoles O_2_ 10^-6^ cells in iso-, hyper-, and hyperosmotic-acclimated cells, respectively, **Figure 2B**). Furthermore, the concomitant increase of both osmosis and HP boosted respiration by a factor of 50 (from 13 to ∼600 nmoles O_2_ 10^-6^ cells), with acclimation not bringing further improvement (639 ± 197 nmoles O_2_ 10^-6^ cells, **Figure 2B**). Hence, hyperosmosis induced a higher respiration activity with respect to hyposmosis. In particular, increased HP resulted in an increased O_2_ consumption by the cells in each treatment.

### Hyposmosis Improves Cell Integrity at Ambient and Increased HP

**FIGURE 3:**
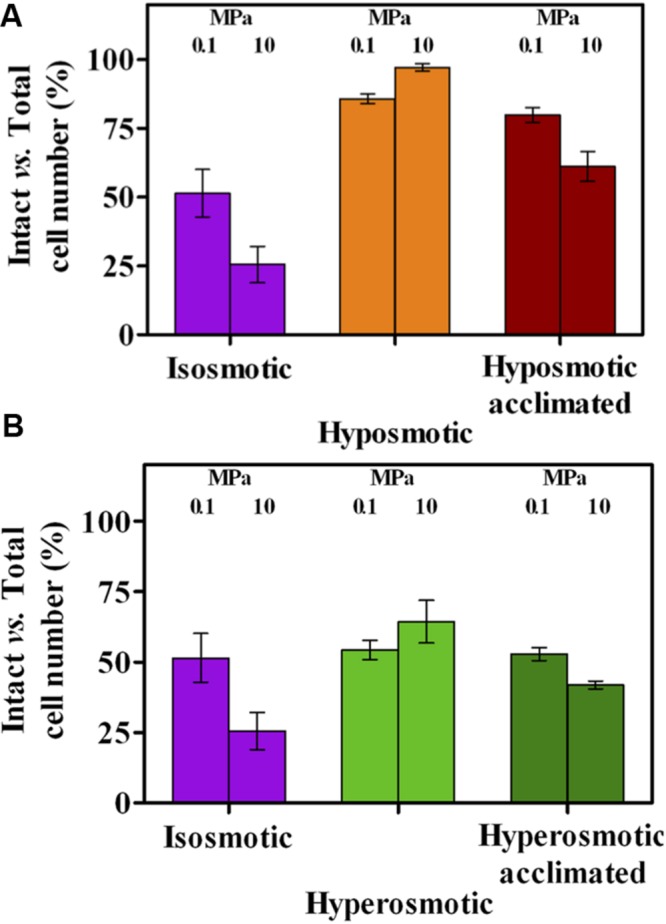
**Relative number of intact cells over total cell count in *A. borkumensis* SK2 cells subjected to hyposmotic **(A)** or hyperosmotic **(B)** conditions under different hydrostatic pressures (0.1 or 10 MPa), as compared to standard isosmotic conditions.** Keys: isosmosis (purple), hyposmosis (orange), hyposmosis acclimated (red), hyperosmosis (light green), hyperosmosis acclimated (dark green). Bars indicate 95% confidence intervals.

Changes in osmosis and HP with respect to standard conditions had a remarkable impact on cell membrane integrity (**Figure 3**). Increase to 10 MPa at isosmotic conditions resulted in a reduced fraction of intact cells (from ∼50 to ∼25%, **Figure 3A**). When growing cells in a medium with reduced osmolarity membrane integrity approached 100%, irrespective of the HP applied (**Figure 3A**), with acclimation slightly reducing these values (**Figure 3A**). At increased osmolarity, no difference was observed at 0.1 MPa even after acclimation (**Figure 3B**), while cell integrity improved significantly when concomitantly increasing both osmolarity and HP, values slightly decreasing after acclimation. As a whole, transition from isosmosis to both altered osmotic conditions conferred enhanced cell integrity under HP (*p* < 0.0001) and, notably, resulted into a higher intact cell number in the most unfavorable condition (i.e., 10 rather than 0.1 MPa). Hence, hyposmosis conferred resistance to HP through structurally resistant cells (**Figure 3A**) with low activity (**Figures 1A** and **2A**), while hyperosmosis counteracted HP by remarkably enhancing metabolic activity (**Figures 1B** and **2B**) while increasing cell integrity to a lower extent (**Figure 3B**).

**FIGURE 4:**
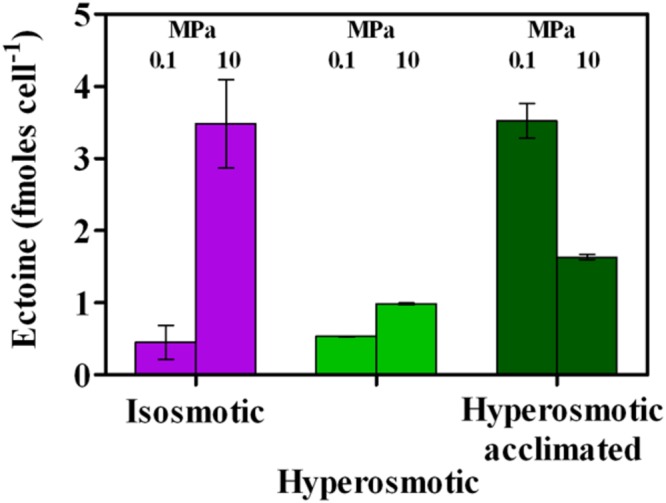
**Ectoine production per cell in *A. borkumensis* SK2 cells subjected to hyperosmotic conditions under different hydrostatic pressures (0.1 or 10 MPa) as compared to standard isosmotic conditions.** Keys: isosmosis (purple), hyperosmosis (light green), hyperosmosis acclimated (dark green). Bars indicate 95% confidence intervals.

Production of the piezolyte ectoine in SK2 cells was substantially different. Under isosmotic conditions, minor amounts of ectoine were produced, with a sharp increase as HP was brought to 10 MPa (**Figure 4**). No trace of ectoine could be detected irrespective of the applied HP or acclimation under hyposmosis (data not shown, detection limit 4 mg L^-1^), suggesting that the remarkable cell integrity (**Figure 3A**) likely relied on a water-reclamation effect. However, cells adapted to hyposmosis did not lose their capacity to produce ectoine, as 4-days incubations of these cells under HP and hyperosmosis did yield some ectoine (Supplementary Figure S1). On the other hand, hyperosmosis at 0.1 MPa led to an increase in the amount of ectoine per cell only by means of acclimation (from 0.5 to 3.5 fmoles ectoine cell^-1^, **Figure 4**), while a different trend was noted with the concomitant increase of osmolarity and HP. As a matter of fact, the highest value was observed in isosmosis by only increasing HP to 10 MPa (3.5 ± 1.1 fmoles cell^-1^, **Figure 4**). The concomitant increase of osmolarity and HP actually reduced ectoine production, which further slightly increased with cell acclimation to high osmolarity (0.98 ± 0.03 and 1.63 ± 0.08 fmoles cell^-1^, respectively, **Figure 4**). This result suggests that *A. borkumensis* cells may use ion transport to counteract HP effects, rather than synthesizing ectoine *de novo*. Further, it should be noted that an increase to 10 MPa HP under isosmosis induced the same ectoine accumulation as after acclimation to hyperosmosis at ambient pressure (**Figure 4**).

### Osmolarity Shocks Impair Culture Performance

**FIGURE 5:**
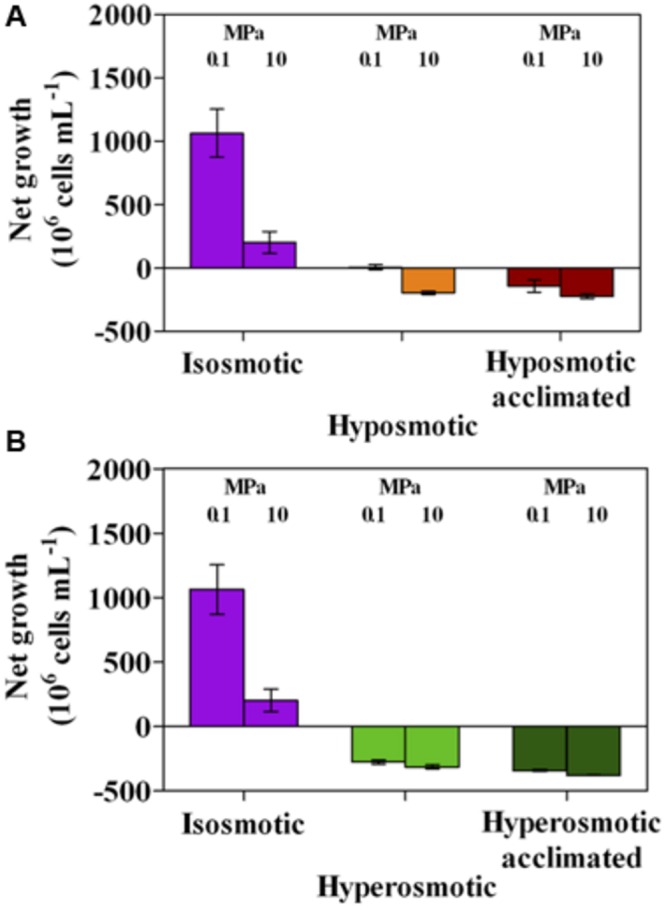
**Net growth in *A. borkumensis* SK2 cultures subjected to hyposmotic **(A)** or hyperosmotic **(B)** conditions under different hydrostatic pressures (0.1 or 10 MPa), as compared to standard isosmotic conditions.** Keys: isosmosis (purple), hyposmosis (orange), hyposmosis acclimated (red), hyperosmosis (light green), hyperosmosis acclimated (dark green). Bars indicate 95% confidence intervals.

Standard conditions of osmosis and HP (isosmosis using the standard ONR7a medium and 0.1 MPa) ideal to *A. borkumensis* SK2 (Yakimov et al., 1998) resulted into a sustained cell replication, which significantly (*p* < 0.05) decreased when cultures were grown under a HP of 10 MPa (**Figures 5A,B**). Incubation at altered osmotic conditions had a detrimental effect on culture growth, as a net decrease in cell number was always observed irrespective of the HP applied (up to -314 × 10^6^ cells mL^-1^; **Figures 5A,B**). Acclimation to different osmolarities yielded similar results, with cultures acclimated to hyperosmosis being the most affected (-375 × 10^6^ cells mL^-1^; **Figure 5B**).

**FIGURE 6:**
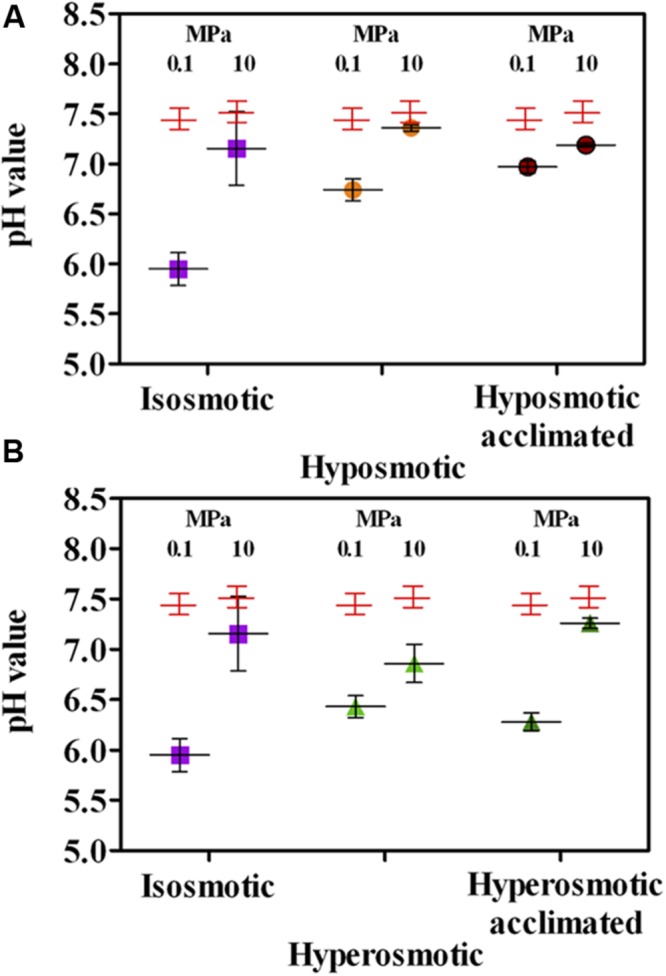
**Changes in pH value in *A. borkumensis* SK2 cells subjected to hyposmotic **(A)** or hyperosmotic **(B)** conditions under different hydrostatic pressures (0.1 or 10 MPa), as compared to standard isosmotic conditions.** Keys: isosmosis (purple squares), hyposmosis (orange circles), hyposmosis acclimated (red circles), hyperosmosis (light green triangles), hyperosmosis acclimated (dark green triangles). Red crosses indicate pH in sterile controls. Bars indicate 95% confidence intervals.

Degradation of *n*-dodecane leading to different CO_2_ production values was consistent with the general decrease in pH value in all conditions (**Figure 6**), as different acidification levels were the combined result of reduced final cell numbers (**Figure 5**) and CO_2_ production per cell (**Figure 1**). Isosmosis and ambient pressure resulted in the lowest pH value (5.95, **Figures 6A,B**), meaning that cells were very active in oil degradation leading to CO_2_ production. In agreement with the observed high activity of cells exposed to hyperosmosis (**Figure 1B**), the pH value was generally lower when osmolarity was increased (6.43 vs. 6.74 at 0.1 MPa in hyper vs. hypo-osmotic cultures, **Figures 6A,B**). Enhanced acidification was not observed in cultures incubated under HP. The final pH in these cultures was always higher than their respective incubation test carried out at 0.1 MPa (6.86 vs. 7.36 at 10 MPa in hyper vs. hypo-osmotic cultures, **Figures 6A,B**), although it can be assumed that under increased HP cells experienced somewhat lower pH values than that measured after decompression.

**FIGURE 7:**
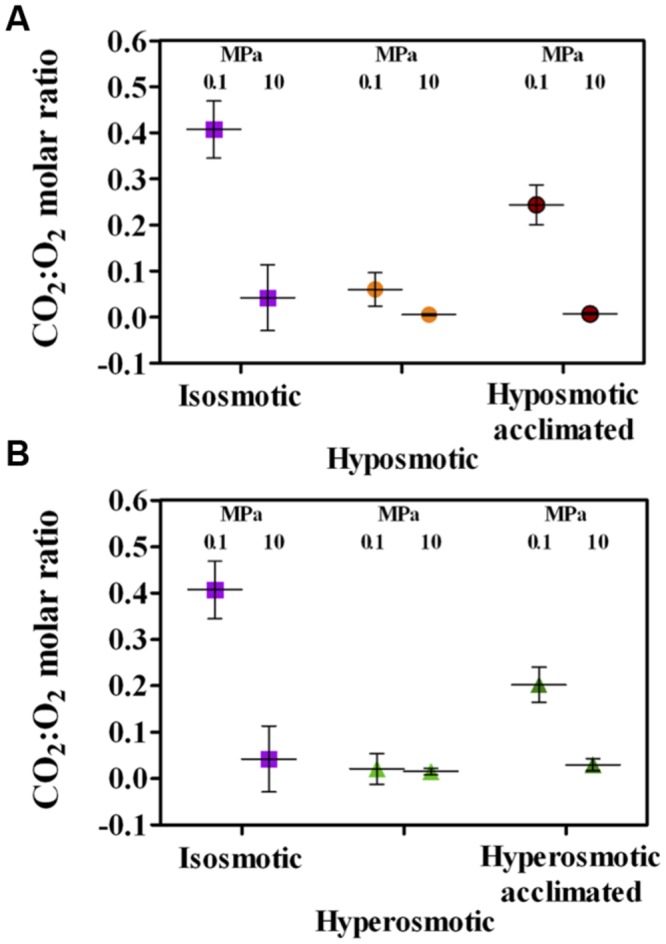
**Molar ratio between CO_2_ production and O_2_ respiration in *A. borkumensis* SK2 cultures subjected to hyposmotic **(A)** or hyperosmotic **(B)** conditions under different hydrostatic pressures (0.1 or 10 MPa), as compared to standard isosmotic conditions.** Note that stoichiometric mineralization of *n*-dodecane would yield a CO_2_:O_2_ ratio equal to 0.649. Keys: isosmosis (purple squares), hyposmosis (orange circles), hyposmosis acclimated (red circles), hyperosmosis (light green triangles), hyperosmosis acclimated (dark green triangles). Bars indicate 95% confidence intervals.

Reduced oil degradation by cultures subjected to osmotic and HP was reflected by a reduced molar ratio between CO_2_ produced and O_2_ respired, compared to the stoichiometric CO_2_:O_2_ ratio for *n*-dodecane oxidation equal to 0.649 (i.e., at least 18.5 moles of O_2_ are needed to oxidize 1 mole of *n*-dodecane, yielding 12 moles of CO_2_). The best efficiency was observed in isosmosis at ambient pressure (0.407, **Figures 7A,B**). Almost all the other degradation efficiencies decreased by a factor of 10 to ∼0.05 or lower, excepting for cultures acclimated to both altered osmotic conditions under ambient pressure, where CO_2_:O_2_ ratio reached ∼0.21 (**Figures 7A,B**). Hence, the effects exerted by changes in osmosis under HP on cell metabolism and integrity (**Figures 1–4**) were not reflected in improved culture performance in terms of cell growth (**Figure 5**) or substrate degradation efficiency (**Figures 6** and **7**).

## Discussion

Improved microbial tolerance to HP consistent with a high resistance to other extreme conditions has been widely reported in the literature, especially with respect to food preservation technology (De Angelis and Gobbetti, 2004). HP-resistant *Escherichia coli* cells were found to be also more resistant to low pH, mild heat, oxidation, and osmotic stress compared to HP-sensitive *E. coli* strains (Benito et al., 1999). Preincubation of *Lactobacillus sanfranciscensis* under increased salinity, low temperature or pH improved its tolerance to HP (Scheyhing et al., 2004). Similarly, HP inactivation of *Lactococcus lactis* was less effective when cells were concomitantly exposed to increased osmolarity (Molina-Höppner et al., 2004). Synergistic effects between these parameters (e.g., pH, temperature, HP) are typically found in bacteria inhabiting environmental niches such as deep-sea hydrothermal vents (Kaye and Baross, 2004). The reasons for this interdependency rely on the fact that at extreme levels some of these conditions may impose a similar physicochemical stress on cell metabolism. HP influences pH_i_ by enhancing the dissociation of weak organic acids and increasing the permeability of the cytoplasmic membrane, which limits the level of pH homeostasis (De Angelis and Gobbetti, 2004), and may resemble the impact of extreme extracellular pH. Bacteria have evolved a number of strategies to counteract the low cell membrane fluidity due to cold temperatures (Chintalapati et al., 2004), a condition also imposed by an increase in HP (Bartlett, 2002). However, the main effect of HP application is volume change, meaning that increased HP accelerates any reaction that entails a reduction in volume, while the opposite is true for a positive volume change (Abe, 2007). This typically impairs cell division and replication (Bartlett, 2002; Abe, 2007) and the activity of several multi-component protein complexes (Oger and Jebbar, 2010), where assemblage of different subunits yields an increase in volume, as in the case of ribosomes and protein translation (Schwarz and Landau, 1972). Water stress- as that following a change in osmolarity in the environment- exerts similar effects on cell metabolism. Hyperosmosis results in a net water eﬄux from the cell, with detrimental loss of cell turgor pressure and cell shrinkage, while a reduced environmental osmolarity leads to a massive influx of water into the cell (Csonka, 1989). In both cases, cells have to quickly react to prevent cell lysis. Rapid shifts in cellular pressure and volume will impact protein–protein and protein–ligand interactions (Heremans, 1982) and compromise enzyme functionality.

In *A. borkumensis* SK2, osmolarity shocks could activate some resistance mechanisms against HP stress, particularly at hyperosmosis where an increased cell metabolism was observed (**Figures 1B** and **2B**), while hyposmosis only impacted O_2_ respiration capacity (**Figure 2A**). The most remarkable effect was exerted on cell integrity, which increased under HP in both altered osmotic conditions with respect to standard isosmosis under HP (**Figures 3A,B**). A positive correlation between reduced cell damage, cell viability and HP resistance has been previously suggested (Benito et al., 1999; Pagán and Mackey, 2000). As it stands, hyposmosis counteracted HP by conferring a high membrane integrity but low activity, while hyperosmosis conferred resistance to HP by strongly improving cell metabolism and enhancing cell integrity to a lower extent.

How do cells counterbalance a shift in osmolarity? Common strategies to maintain homeostasis are intracellular accumulation of organic compounds (by the bacterial class of “osmoconformers”) and/or ion transport (by the “osmoregulators”), being the choice between the two based on evolutionary and, particularly, energetic aspects (Oren, 1999). Cell membranes are permeable to water, and rely on eﬄux or influx/production of such osmolytes to counterbalance water stress. The average concentration of salts in seawater brings about 1000 mOsm kg^-1^ (mainly due to NaCl), which is 2–3 times more than that found in most cells (∼300–400 mOsm kg^-1^; Yancey, 2005). In the present study, isosmotic conditions were equivalent to 1136 mOsm kg^-1^, while hypo- and hyperosmotic were 330 and 1720 mOsm kg^-1^, respectively (**Table 1**). Hence, *A. borkumensis* SK2 cells grown in hypo-, iso-, and hyperosmosis experienced salinities equivalent to brackish, saline and brine waters, respectively (**Table 1**). Strain SK2 was isolated from sea water and sediment samples collected near the Isle of Borkum (North Sea), under ambient pressure. It grows at salinities comparable to other heterotrophic marine halophilic γ-Proteobacteria with which it shares many traits such as *Marinobacter, Oceanospirillum*, and *Halomonas* (between 3 and 10% NaCl, Yakimov et al., 1998). For instance, the hydrocarbonoclastic *Marinobacter* and *Alcanivorax* genera were detected in oil mousses (ambient pressure) 1 year after the DWH spill, but their relative abundance was reduced in salt marshes (Liu and Liu, 2013). Hence, the present hyperosmotic condition was within the living boundaries for SK2, while hyposmosis must have exerted a stronger stress. Conversely, *Alcanivorax* presence was low and not correlated with hydrocarbon levels in DWH sediment samples (Kimes et al., 2013) and its contribution in the DWH oil plume at deep-sea considered negligible (Gutierrez et al., 2013). Together with low temperature (Gutierrez et al., 2013), HP has been proposed as a major driver to explain *Alcanivorax* absence in the deep sea (Scoma et al., 2016). Recent findings on strain SK2 subjected to 10 MPa in standard marine media reported a remarkable accumulation of ectoine, a cyclic, amino acid derivate organic osmolyte which has now been proposed as a piezolyte, as it also accumulates as a result of increased HP (Scoma et al., unpublished results). The capacity of several organic osmolytes to engage in unique reactions exerting a protective effect on cells in ways other than *via* osmotic-based strategies supported the hypothesis that some solutes might in fact have a role also in counteracting HP. Previous examples are *N*-trimethylamine oxide (TMAO; reviewed by Yancey, 2005) and probably β-hydroxybutyrate (Martin et al., 2002).

In the present study, estimates of ectoine accumulation on a cell dry weight (CDW) basis were of the same order of the highest found in literature at ambient pressure (up to 0.5 g_ectoine_/g_CDW_, Supplementary Table S1). However, owe to a net decrease in cell number (**Figure 5**), ectoine final concentration in the broth medium was rather low (<0.14 g L^-1^, Supplementary Table S1). The highest (comparable) productivities were stimulated by high HP or salinity, that is isosmosis at 10 MPa or hypersomosis at ambient pressure (**Figure 4**), although cells exposed to HP were incubated for 4 days while acclimation to hyperosmosis lasted for ∼30 days.

The mechanisms by which ectoine might offset HP are unknown, hence its classification as compatible, counteracting or compensatory solute remains unclear (Yancey, 2005). The complete lack of ectoine production consistent with a decreased metabolic activity in hyposmosis indicate that this piezolyte might be involved to some extent in maintaining functional cellular processes. However, its concentration (**Figure 4**) was not linearly related with any cell metabolic activity evaluated in this study (**Figures 1, 2, 6**, and **7**). In halophilic bacteria such as *Alcanivorax* ectoine functions as a water-reclaimer preventing dehydration has been widely assessed, owe to its capacity to form water structures in its proximity (da Costa et al., 1998). In non-halophilic streptomycetes it was also observed to protect antibiotic-producing cells from their own products (da Costa et al., 1998). Possibly, ectoine functionality as a piezolyte may still be to act as water-reclaimer just as osmolytes (reviewed in da Costa et al., 1998; Yancey, 2005). In this respect, it must be noted that the water-reclaming, ectoine-free cell membrane protection to HP observed in hyposmosis was almost complete (**Figure 3**). This hypothesis needs further experimental evidence. Notably, concomitant application of high osmolarity and HP did not sum up to yield even higher ectoine productivities as compared to isosmosis at 10 MPa but resulted into lower ectoine concentrations (**Figure 4**). This suggests that, when possible (e.g., in hyperosmosis), in *A. borkumensis* SK2 ion transport may be concomitantly used as an energy-efficient mechanism to counteract HP (Oren, 1999). Under isosmosis cells may have no other strategy to counteract HP other than synthesizing ectoine, while environmental hyperosmosis may still be regulated by the cell through ion transport. Notably, the energy-intensive ectoine production (Bernard et al., 1993) does not bring any growth advantage to SK2 under HP (**Figure 5**). The lack of a linear ectoine accumulation following osmotic shocks suggests that resistance to HP through an unbalanced osmolarity is achieved through different strategies, which may compensate each other to some extent. Nonetheless, ectoine synthesis under HP can be predicted in other halophiles closely related to *Alcanivorax* and possessing the *ectABC* gene cluster such as *Halomonas*.

Notwithstanding the positive effects observed on cell activity and integrity, osmotic shocks always negatively impacted culture performance at HP. No net culture growth was observed during the 4-days incubation test (**Figures 5A,B**), and growth was only observed by growing cultures for 7–10 days (as during the acclimation phase). Medium acidification resulting from CO_2_ production was severely impacted (**Figures 6A,B**), and mirrored in the reduced efficiency in *n*-dodecane utilization (**Figures 7A,B**), which dropped to ∼0.05 CO_2_:O_2_ ratio under HP irrespective of the osmolarity applied. These results confirm the piezosensitive nature of *A. borkumensis* SK2, a model organism for hydrocarbonoclastic bacteria, which is highly impaired by a mild increase in HP (Scoma et al., unpublished results). While dominating bacterial blooms in oil-polluted surface waters worldwide (Yakimov et al., 2005), in deep-sea contaminated waters *A. borkumensis* presence is negligible (Hazen et al., 2010; Valentine et al., 2010; Baelum et al., 2012; Mason et al., 2012; Yergeau et al., 2015). Osmotic shocks may partially cope with HP stressing effects, but their main outcome is to support survival rather than growth, highlighting the lack of proper HP adaptation mechanism in *A. borkumensis* SK2. Similar responses might be descriptive of other piezosensitive bacteria in the water column, and contribute to shape microbial communities in oil-contaminated deep-sea environments.

## Author Contributions

AS conceived the project, designed and performed the experiments, analyzed the data, and wrote the paper. NB conceived, supervised, and funded the project.

## Conflict of Interest Statement

The authors declare that the research was conducted in the absence of any commercial or financial relationships that could be construed as a potential conflict of interest.
