# Supplementary material for: Osmotic Stress Confers Enhanced Cell Integrity to Hydrostatic Pressure but Impairs Growth in Alcanivorax borkumensis SK2
Source: Front Microbiol. 2016 May 18;7:729. doi: 10.3389/fmicb.2016.00729 (PMC4870253; doi:10.3389/fmicb.2016.00729)
Supplement: Supplementary file 1 [file Table_1.DOC]

**Table 1. Comparative ectoine productivity in *A. borkumensis* SK2 in the present study as compared to previous investigations.** Biomass yields in terms of grams of cells dry weight (gCDW) were estimated using the final cell numbers at the end of the incubations and the average cell weight of 10^-12^ gCDW/cell (**Davis et al**., 1973, in “*Bacterial physiology: microbiology*”, pp 96-97, Eds. Harper and Row, Maryland; **Loferer-Krößbacher et al.**, 1998, *Determination of bacterial cell dry mass by transmission electron microscopy and densitometric image analysis.* *Applied and Environmental Microbiology 64*(2): 688-694).

| **Strain** | **Carbon source** | **Osmotic pressur**e | | **HP** | **Ectoine accumulation yield**s | |
| --- | --- | --- | --- | --- | --- | --- |
|  |  | **Condition** | **NaCl [M]** | **MPa** | **g_ectoine_/g_CDW_** | **g L^-1^** |
| *A.borkumensis SK2* (this work) | *n*-dodecane | Isosmotic | 0.4 | 0.1 | 0.06 | 0.07 |
|  |  |  |  | 10 | 0.49 | 0.10 |
|  |  | Hyperosmotic | 0.8 | 0.1 | 0.08 | 0.01 |
|  |  |  |  | 10 | 0.14 | 0.01 |
|  |  | Hyperosmotic adapted |  | 0.1 | 0.50 | 0.03 |
|  |  |  |  | 10 | 0.23 | 0.00 |
| *Brevibacter epidermis* (Onraedt et al., 2004) | Yeast extract | n.d. | 1.0 |  | 0.14 | 0.98 |
|  |  |  | 1 | 0.1 | 0.16 | 8.00 |
| *B. epidermis* (Onraedt et al., 2005) | Rich medium |  |  |  |  |  |
|  |  |  | 1.0 | 0.1 | 0.21 | 0.19 |
| *B. linens* (Bernard et al., 1993) | Lactate |  |  |  |  |  |
|  |  |  | 2.0 | 0.1 | 0.17 | 0.80 |
|  |  |  | 0.7 | 0.1 | 0.07 | 4.30 |
| *Brevibacterium sp.* (Nagata and Wang, 2001) | Rich medium |  |  |  |  |  |
|  |  |  | 0.51 | 0.1 | 0.06 | n.d. |
| *Halomonas boliviensis*  (Guzman et al., 2009) | Rich medium |  |  |  |  |  |
|  |  |  | 0.5 | 0.1 | 0.35 | 6.90 |
| *E. coli DH5 α* (Bestvater et al., 2008) | Rich medium |  |  |  |  |  |
| *H. salina* (Zhang et al., 2009) | Monosodium glutamate |  |  |  |  |  |
