# Supplementary figures and images for: Osmotic Stress Confers Enhanced Cell Integrity to Hydrostatic Pressure but Impairs Growth in Alcanivorax borkumensis SK2
Source: Front Microbiol. 2016 May 18;7:729. doi: 10.3389/fmicb.2016.00729 (PMC4870253; doi:10.3389/fmicb.2016.00729)

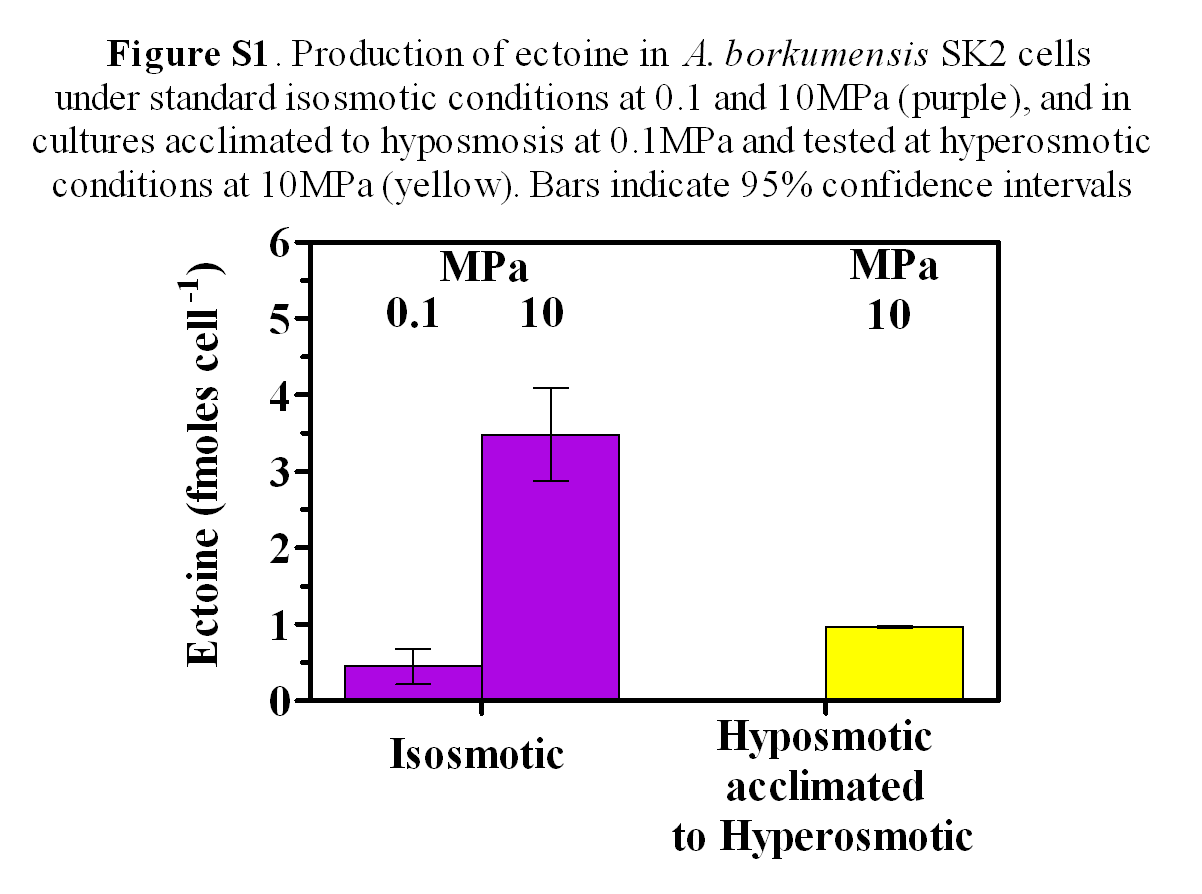

Supplement: Supplementary file 2 [file Image_1.TIF]
